# Will vaccination against rotavirus infection with RIX4414 be cost-saving in Germany?

**DOI:** 10.1186/2191-1991-3-27

**Published:** 2013-11-18

**Authors:** Stefanie Knoll, Christoph Mair, Ursula Benter, Katja Vouk, Baudouin Standaert

**Affiliations:** 1 GlaxoSmithKline GmbH & Co. KG, Munich, Germany; 2 INC Research GmbH, Munich, Germany; 3 GlaxoSmithKline Vaccines, Wavre, Belgium

**Keywords:** Cost savings, Economic evaluation, Germany, Paediatric, RIX4414, Rotavirus, Vaccination

## Abstract

**Background:**

Rotavirus gastroenteritis (RVGE) is a frequent disease in young children. The recommended German paediatric immunisation schedule does not currently include rotavirus vaccination. A lack of economic data on the impact of routine vaccination is stated as one of the reasons. As a result, the current coverage rate is low, around 26%. This study investigated whether rotavirus vaccination using the two-dose rotavirus vaccine RIX4414 (*Rotarix*®, GlaxoSmithKline Vaccines) would be a cost-saving intervention from the perspective of the statutory health insurance (SHI) in Germany.

**Objective:**

The objective of the study was to analyse health outcomes (number of RVGE cases and hospitalisations prevented) and the associated cost to the SHI when comparing 100% rotavirus vaccination with no vaccination in Germany.

**Methods:**

A Markov cohort model simulated the number of RVGE events and related costs in a German birth cohort over the first 60 months of life with current disease management. The model compared an unvaccinated cohort with a fully vaccinated cohort. Vaccine efficacy data from international clinical trials were combined with German-specific epidemiological and cost data. Results were tested using extensive sensitivity analyses.

**Results:**

Full vaccination of a birth cohort against rotavirus disease would be expected to prevent 82% of RVGE cases, reducing RVGE frequency from 28 to 5 events per 100 children in the birth cohort up to age 5 years. The estimated cost reduction with vaccination for that period is predicted to be €9.2 million with 100% coverage (€6.9 million with 75% coverage), mainly due to reductions in SHI reimbursement for productivity losses, hospital stays and visits to office-based physicians due to the vaccine’s efficacy against severe disease.

**Conclusions:**

Routine rotavirus vaccination in Germany would reduce the number of hospitalised and outpatient cases. The associated investment could be fully offset by costs avoided in hospital stays, physician visits and SHI reimbursement of productivity losses. Sensitivity analysis indicated that vaccination would be cost-saving in 95% of simulations. Incremental cost was observed only under extreme conditions, especially when the time spent at home due to rotavirus disease was low or when vaccine efficacy against severe disease was heavily decreased.

## Background

Rotavirus is the leading cause of acute gastroenteritis (AGE) in young children [1,2]. The virus is highly contagious and is usually transmitted by the faecal-oral route [3]. Seventy per cent of children are infected at least once before the age of 5 years [4-6], and a much lower proportion are infected three to four times over the same age range. The first infection is the most severe, especially when it occurs at a very young age when natural immunity is not well developed [6]. The disease burden of rotavirus gastroenteritis (RVGE) arises from the combination of a high incidence rate, severity of symptoms leading to hospitalisation (particularly in very young infants), and the consequent distress caused to children and other family members.

In the European Union rotavirus is estimated to cause more than 200 deaths each year, over 87,000 hospital admissions, and almost 700,000 outpatient visits in children under the age of 5 years [7]. The burden of the disease in Germany is well documented through epidemiological surveys [1,8-10], cost-of-illness studies [11,12], and a family impact study [13]. According to the Robert Koch Institute, RVGE is the most frequent registered disease in children aged <5 years in Germany, with over 612,000 reported rotavirus infections since 2011 [14].

Two oral rotavirus vaccines are currently available for the prevention of rotavirus infection in Germany: a two-dose monovalent human rotavirus vaccine RIX4414 (*Rotarix*®)^a^ and a three-dose pentavalent bovine-derived vaccine (*RotaTeq*™)^b^, both available since 2006. The oral live attenuated human rotavirus vaccine RIX4414 contains only one strain, G1P[8]. Two doses of the vaccine have been shown to be highly immunogenic, well-tolerated and protective against RVGE [15-18].

Several German federal states have already issued recommendations for rotavirus vaccination and a number of statutory health insurance (SHI) funds grant reimbursement for rotavirus vaccination [19]. However, rotavirus vaccination is not part of the federal German recommended immunisation schedule. As a result, the overall vaccination coverage rate is low with large regional variation [20]. The coverage rate among children aged less than 2 years was only 26% in 2010 [20].

The economic value of rotavirus vaccination has been investigated in many countries in the developed world, but there is controversy about the conclusions of the analyses [21]. The lack of country-specific economic analysis from the SHI perspective for Germany has been stated by the Ständige Impfkommission (Standing Committee on Vaccination [STIKO]) of the Robert Koch Institute as one reason why the vaccine is not included on the list of recommended vaccinations for children^c^.

Earlier evaluations have shown that rotavirus vaccination in Germany could be a cost-saving intervention from a societal perspective, but only very limited data are available from the SHI perspective [22-24]. The analysis presented here evaluates the impact of rotavirus vaccination on healthcare resource use and the associated economic impact from the perspective of a third-party payer. In Germany, incremental cost-effectiveness ratios (ICERs) are not used in economic evaluations of pharmaceuticals, nor are they established as standardised thresholds for recommendations by STIKO. Therefore we have presented health outcomes (RVGE cases prevented) and cost results (cost savings) separately, without any attempt to estimate a cost-effectiveness ratio.

## Methods

### Markov model

**Figure 1:**
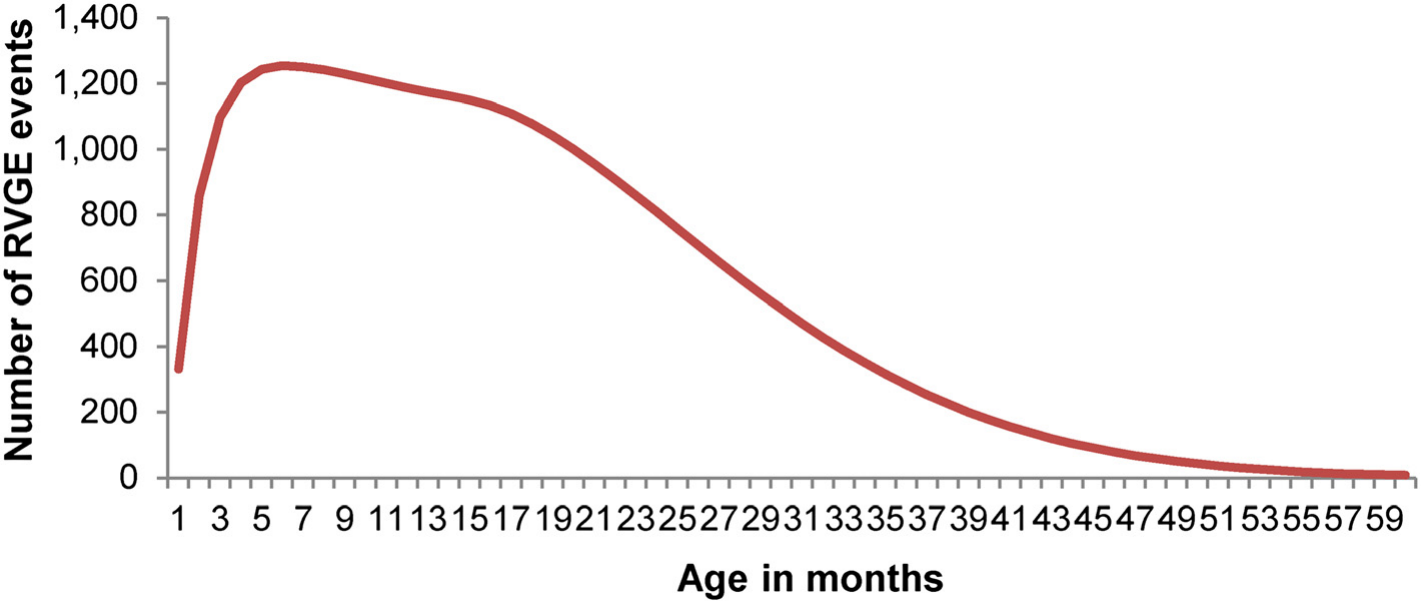
**Rotavirus diarrhoea episodes as a function of age.** Distribution of rotavirus diarrhoea episodes as a function of age in the German birth cohort of 2008.

A *Microsoft Excel*-based Markov cohort model has been used to compare predicted outcomes and costs between full vaccination (100% coverage) and no vaccination. The model has been described previously and reported elsewhere [25]. It follows the German birth cohort of 2008 (n = 682,514) until the cohort reaches 5 years of age, with a time cycle of one month (60 cycles in total). It has been adapted to the German natural death rate for children aged 0–5 years and to German treatment pathways. In each monthly cycle the cohort is exposed to the risk of experiencing a first episode of rotavirus diarrhoea. The risk is age-dependent over the age range modelled (0–5 years) and follows a Weibull distribution (coefficients 1.5 for the shape and 24.2 for the scale), with a shift to the right ending in a long tail (Figure 1).

An episode of RVGE may result in a visit to a medical practitioner in an office-based setting, hospitalisation (i.e. a visit to the emergency room followed by hospitalisation), or staying at home and watchful waiting without consulting a medical practitioner. Visits to office-based physicians can be followed by hospitalisation. This is consistent with the findings of the REVEAL study, in which 33-68% of children with AGE who first presented to primary care subsequently required additional medical care in another setting [26]. Informal care at home after hospitalisation does not require a further visit to an office-based physician. During hospitalisation, death may occur due to RVGE.

Any child aged under 5 years may be exposed to another RVGE after the first episode. Repeated rotavirus infections have been shown to induce progressive natural immunity that protects against subsequent infections [6], and therefore second and subsequent infections are unlikely to be severe. Thus we assumed that the second RVGE event can be treated by an office-based physician and never leads to an emergency visit or hospitalisation.

During the first months of life a reduced number of RVGE episodes is observed, compared with older infants and children, as a result of protection conferred by circulating maternal antibodies [27-29]. However, as they are unprotected by natural immunity, children hospitalised for a non-rotavirus-related event are at risk of acquiring nosocomial rotavirus infection. The model accounts for both these aspects.

The model does not account for any herd effect from vaccination.

### Model input parameters

#### Epidemiological data

This analysis compares 100% vaccination with no vaccination. To assess the no-vaccination condition, the model has been populated with epidemiological data reflecting the German situation in 2008 when the rotavirus vaccine coverage rate was very low, no more than 10%. The input data are summarised in Table 1. Model transition probabilities as a function of age for monthly time units are generated by calibrating the model to fit German data for numbers of community-acquired RVGE cases, outpatient physician visits, hospital stays, nosocomial RVGE cases, and RVGE-specific deaths. We selected the birth cohort of 2008 as a reference [30] instead of an average over several years in order to allow for the decreasing trend in number of births over time in Germany, and because 2008 was the last year in which the proportion of rotavirus-vaccinated children was <10%.

**Table 1:** Epidemiological data for a birth cohort of 682,514 infants

**Parameter**	**Proportion of birth cohort**	**Number of cases**	**Source**
Total RVGE cases	28.10%	192,037	Calculated
Untreated RVGE cases	6.20%	42,492	Calculated
Outpatient-treated RVGE cases	21.90%	149,544	[10]
Hospital-treated RVGE cases	4.93%	33,648	Calculated [9,10]
Nosocomial RVGE cases	0.41%	2 795	Calculated from different sources [31-33]
Deaths		1	[34]

*RVGE* rotavirus gastroenteritis.

The initial data inputs for the model were entered as a proportion of the birth cohort exposed to the risk of developing rotavirus diarrhoea for the time period from birth to the age of 5 years. For example, approximately 29% of the birth cohort will experience rotavirus diarrhoea before the age of 5 years, and the distribution of these disease episodes as a function of age follows the curve shown in Figure 1. A similar exercise was performed for medical visits, hospitalisations, nosocomial RVGE, and RVGE-specific deaths.

The estimated proportion of the birth cohort developing RVGE between the age of 0 and 5 years in Germany is the sum of the proportion attending primary care settings for RVGE plus the proportion of parents who do not take their child suffering from any kind of diarrhoea to a physician.

Based on the annual number of AGE cases (Diagnosis-related group [DRG]: A08.0, A08.4 and A09) in the Federal Health Report in the age group 0–5 years between 2000 and 2008 [35], the average proportion of the birth cohort hospitalised for an AGE event is 8.65%. As AGE cases are not routinely tested for rotavirus, the reported number of RVGE cases should be seen as an underestimate. The reported numbers result in an annual incidence of 1.10 per 100,000 children aged 0–5 years, while other studies report much higher country-specific annual incidence rates, up to 4.680 per 100,000 [1,8-10,14]. The REVEAL [10] and SHRIK [9] studies reported that RVGE infections accounted for up to 66% of all hospitalised AGE cases. In the absence of reliable reported data, we used these numbers to estimate RVGE cases. In the base case we assumed that RVGE cases accounted for 57% instead of 66%. The range tested in sensitivity analysis was from 42% to 66%.

The available data for nosocomial RVGE events have some limitations, as the studies are either based on reported cases only or are derived from a single centre, and provide different estimates for the same age group (between 0 and 5 years old) [31-33]. We selected the lowest estimate, 0.41% of the birth cohort, as the base-case value for nosocomial RVGE.

The Federal Statistical Office of Germany reported one death due to RVGE in the relevant age group in 2006 [34], and that value has been included in the model.

#### Vaccine efficacy data

In the absence of country-specific studies, vaccine efficacy (VE) data for RIX4414 were collected from the double blind, randomised, controlled trial conducted in six European countries that was the basis for the approved Summary of Product Characteristics for *Rotarix*® [36,37]. Jit et al. [38] summarised vaccine efficacy results after a first and second dose for rotavirus diarrhoea of three severity levels: mild (no medical visit), moderate (medical visit), and severe (hospitalisation). These severity levels were also used in the model. Confidence intervals for each VE value were estimated using the technique of Wilson [39], as proposed by Newcombe [40]. They were used in the sensitivity analyses, where the range was from a minimum of 69.4% for VE against mild disease after the first dose to a maximum of 100%. In sensitivity analysis, the model also takes into account the change in VE over time as measured in the European trial.

Using data from a European trial in a model for Germany implicitly assumes that the rotavirus genotype distributions in Germany are comparable with those in the countries in the trial. Although the diversity of rotavirus strains co-circulating in the European population is high, this assumption is appropriate based on a review of the available literature. The published data show that the genotype combinations, distributions and observed trends in Germany are comparable with those in the European region [41-44].

#### Cost-related data

Unit costs were assigned reflecting the perspective of the statutory health insurance (SHI) with a reference year of 2011. Cost inputs are summarised in Table 2. They include productivity losses of parents as far as they are reimbursed by the SHI (70% of net income of each parent from day 1 when the medical event begins until day 9) [45]. Because precise data on the number of parents utilising this option of reimbursement are lacking, SHI reimbursement has been approximated in the model using the average salary of a woman aged 20–35 years in Germany. This can be considered as a conservative approach, as a gender payment gap remains in many countries in Europe, including Germany [46]. No cost from the SHI perspective is considered for untreated cases. The Federal Statistical Office of Germany reports that 28% of mothers with children aged less than 3 years and 55% of those with children aged between 3 and 5 years old were working in 2008 [47]. It is assumed that only families with both parents working would request SHI reimbursement for productivity loss.

**Table 2:** Cost data

**Item**	**Proportion**	**Value**	**Source**
Consultation of office-based physicians		42 €	EBM 2011 codes 3110 and 4110 [48]
Outpatient medication		5 €	Dimenhydrinate and paracetamol, taking into account reductions due to patient co-payments and mandatory rebates [49]
Hospitalisation		1 675 €	G-DRG-System 2011 [50]
Hospital stay for 1 parent (children aged <8 years)		45 €/day	[51]
Nosocomial cases		439 €	G-DRG-System 2011 [50]
SHI-reimbursed productivity loss		78 €/day	70% of average salary for women (aged 20–35 years) [52]
Proportion of families with productivity loss (both parents working)	31%		Average of reported data from 0–3 year age group and 4–5 year age group weighted by the incidence of RVGE in these age groups [47,53]
Days staying at home for an outpatient-treated RVGE		5.3 days	REVEAL study data for Germany [11]
Days staying at home for hospitalised RVGE		5 days	Average length of hospital stay, REVEAL study data for Germany [26]
Vaccine costs per dose		51 €	*Rotarix*® 10-dose package taking into account mandatory rebates [49]
Administration costs per dose		6 €	Regional contracts (e.g. “Barmer GEK”) [54]

*DRG* Diagnosis-related group, *EBM* Einheitlicher Bewertungsmaßstab (Doctors’ fee scale), *RVGE* rotavirus gastroenteritis, *SHI* statutory health insurance.

For the base case we took into account the age-specific probabilities of RVGE in the age group of children less than 5 years old and calculated a weighted average of the proportion of families in which both parents were working (31%). The range tested in sensitivity analyses was 10 to 55%. The duration of hospitalisation for children with RVGE was 5 days [26]. The reported average number of workdays lost was 5.3 days for children with RVGE treated in primary care [11]. As the studies [11,26] provided no confidence interval for these values, we assumed that both the duration of hospitalisation and for staying at home ranged between 2 and 7 days in sensitivity analyses.

Increased duration of hospitalisation due to nosocomial rotavirus infection varied between 1.7 and 5.9 days in the study of Gleizes et al. [55]. The base case evaluation used a value of 2 days, with sensitivity analysis ranging from 1 to 4 days. Data on the number of days at home due to hospitalisation were taken from the Institute for the Hospital Remuneration System [56].

A monthly discount rate was applied to all costs and economic benefits, set at a value that equates to 3% per year [57].

#### Sensitivity analysis

One-way sensitivity analysis was conducted to account for the uncertainty in the data due to a lack of unequivocal reference values. In addition to conducting one-way sensitivity analysis on the cost difference, we also investigated sensitivity using probabilistic sensitivity analyses (PSA) on those variables that indicated an impact on the cost result after performing the one-way sensitivity analysis. This allowed us to limit the number of variables selected for this analysis. PSA results indicate the range over which variables may fluctuate and the probability that vaccination leads to cost savings. Distributions were assigned to the tested variables (Table 3). Some variables have a normal distribution assigned to their spread of values. These are mainly the vaccine efficacy parameters, together with the age-specific incidence rate of diarrhoea. Second-order Monte Carlo simulations were run with *@RISK* software (Palisade, UK, 2011). Up to 5000 iterations were performed to obtain the cost distribution results.

**Table 3:** Variables and their distribution values used in PSA testing

**Variable**	**Distribution type**	**Mean**	**Source**
Average number of days staying at home after consultation visit	General (2,3,4,5,6,7; 0.05,0.05,0.15,0.2,0.3,0.25)	5.2	[11]
Average number of days staying at home due to hospitalisation	General (2,3,4,5,6,7; 0.05,0.02,0.02,0.25,0.25,0.05)	4.6	[56]
Average number of days staying at home due to nosocomial rotavirus infection	Gamma (2,1)	2	[26]
Proportion of families with both parents working	Normal (0.31, 0.031; truncated 0.1,0.55)	31%	[47]
Proportion seeking medical advice	Beta general (6.5,7; 0.65,0.9)	77%	[1]
Probability of hospitalisation	Beta general (10,5; 0.17,0.26)	23%	[9]
Probability of nosocomial infection in the first year	Beta general (6,4; 0.01,0.048)	3.28%	[31-33]
Vaccine efficacy for mild RVGE, 1 year, 1 dose	Normal (0.784; 0.03)	0.784	[36,38]
Vaccine efficacy for moderate RVGE, 1 year, 1 dose	Normal (0.808; 0.03)	0.808	[36,38]
Vaccine efficacy for severe RVGE, 1 year, 1 dose	Normal (0.900; 0.03)	0.900	[36,38]
Vaccine efficacy for mild RVGE, 1 year, 2 doses	Normal (0.871; 0.03)	0.871	[36,38]
Vaccine efficacy for moderate RVGE, 1 year, 2 doses	Normal (0.898; 0.03)	0.898	[36,38]
Vaccine efficacy for severe RVGE, 1 year, 2 doses	Normal (1.000; 0.1; truncated (0.871; 1)	0.94	[36,38]
Age-specific incidence rate of diarrhoea	Normal (0.023; 0.0023)	0.023	
Vaccine coverage rate	Uniform (0.75;1)	87.5%	
Discount rate on cost	Discrete (0,0.75,1,1.5)	3%	

*PSA* probabilistic sensitivity analysis, *RVGE* rotavirus gastroenteritis.

We used regression analysis to evaluate whether the same variables influence the cost-difference results in PSA as in the one-way sensitivity analysis.

## Results

### Base case

The model predicted that full vaccination of the birth cohort of 2008 (n = 682,514) with RIX4414 would have the potential to prevent 134,396 medically treated community-acquired RVGE cases and 22,438 home-treated cases during the first five years of life. This corresponds to a decrease of almost 82% in RVGE cases, from 192,037 (28% of the birth cohort) to 35,203 cases (5%). Consultations with office-based physicians would be expected to decrease from 149,544 (22%) to 15,148 (2.2%), and hospitalisations from 33,648 (5%) to 527 (0.077%). Nosocomial infections would also be expected to decrease, from 2,795 (0.41%) to 238 events (0.035%).

Table 4 shows the cost from the SHI perspective for both scenarios, no vaccination and full vaccination of the birth cohort. Full vaccine coverage would be expected to lead to a potential cost saving of €9.2 million overall for the SHI, when direct medical costs and indirect costs are both included. This is due to the large reduction in RVGE events, leading in turn to a substantial decrease in hospitalisations and consultations with office-based physicians, saving approximately €60 million and €6 million in direct medical costs, respectively, and approximately €64 million and €21 million, respectively, when indirect costs are also included (Table 4). In the cohort with full vaccination, the largest cost segment is the cost of vaccination, at €77.4 million including administration costs. The SHI reimbursement cost for productivity losses incurred by parents represents a large part of the cost, up to €18 million without vaccination and decreasing to €0.9 million with full vaccine coverage. Overall, the cost offsets in reductions in direct medical cost and SHI reimbursement cost for productivity losses would be expected to exceed the cost of vaccination in the base case, thus resulting in a net saving for the SHI. The cost per child at risk was estimated at €–0.63 per year from the SHI perspective.

**Table 4:** Cost impact of vaccination

**Cost item**	**No vaccination**	**Vaccination**	**Difference**
**Direct medical cost only**			
Vaccine cost		€ 77 424 462	€ 77 424 462
Consultation medical visit	€ 6 748 676	€ 686 730	-€ 6 061 946
Hospitalisation	€ 61 456 155	€ 1 000 252	-€ 60 455 903
Nosocomial infection	€ 1 200 255	€ 104 523	-€ 1 095 732
**Total**			**€ 9 810 880**
Direct medical cost plus indirect cost*			
Vaccine cost		€ 77 424 462	€ 77 424 462
Consultation medical visit	€ 23 886 005	€ 2 430 584	-€ 21 455 421
Hospitalisation	€ 65 054 063	€ 1 058 811	-€ 63 995 253
Nosocomial infection	€ 1 330 780	€ 115 890	-€ 1 214 890
**Total**			**-€ 9 241 102**

Impact of vaccination (100% coverage) on direct medical costs and indirect costs from SHI perspective.

*SHI* statutory health insurance.

*Includes SHI reimbursement for productivity losses associated with medical visits, hospitalisations and nosocomial infections.

With a vaccination coverage rate of 75% (at the lower end of coverage rates achieved for STIKO-recommended vaccinations), the projected overall cost saving to the SHI was estimated at €6.9 million.

### Sensitivity analyses

**Figure 2:**
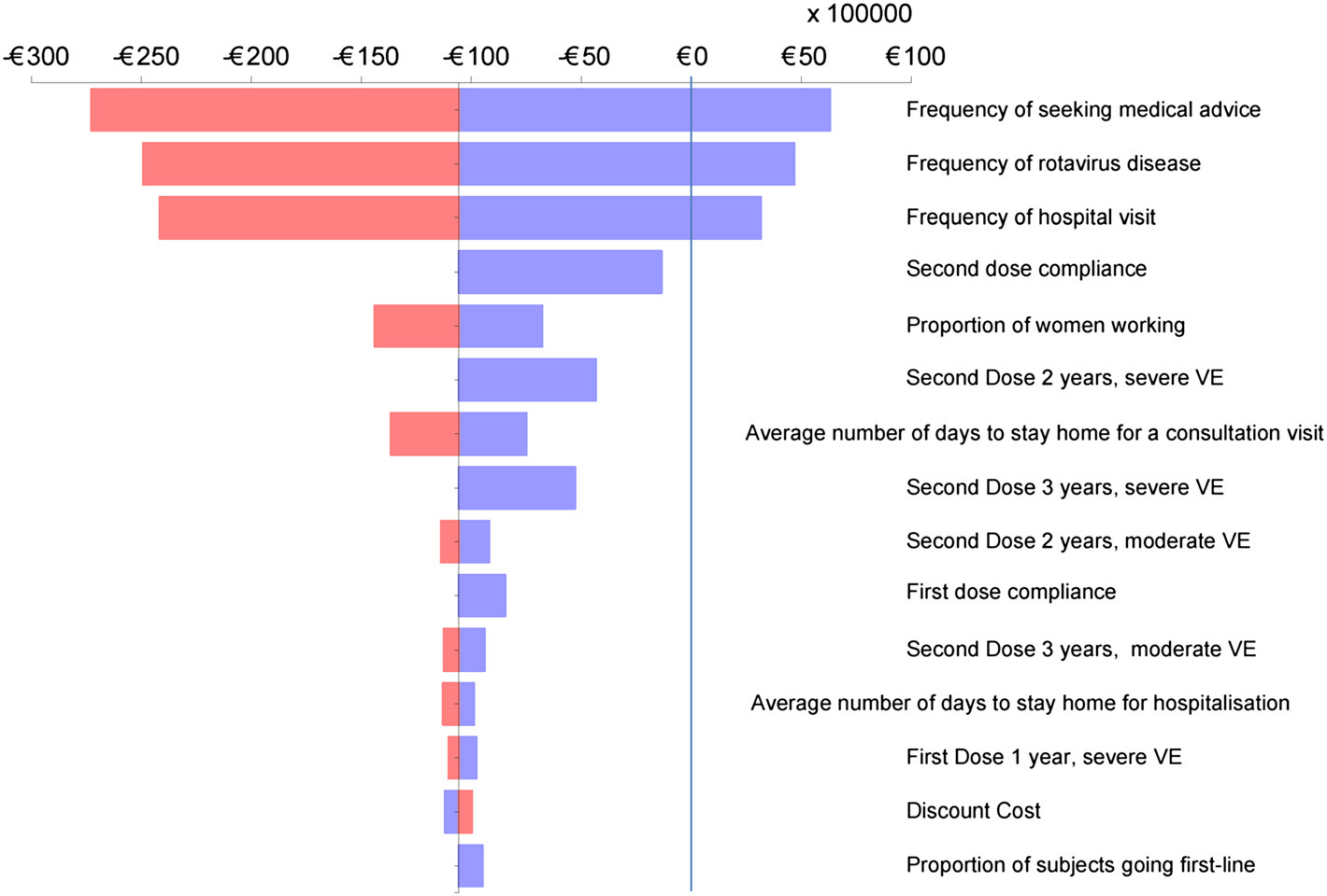
**One-way sensitivity analyses.** Tornado diagram showing results of the one-way sensitivity analyses for the difference (vaccination versus no vaccination) in total cost per vaccinated child. The vertical line indicates where the incremental costs for the vaccine strategy were more than €0.

**Figure 3:**
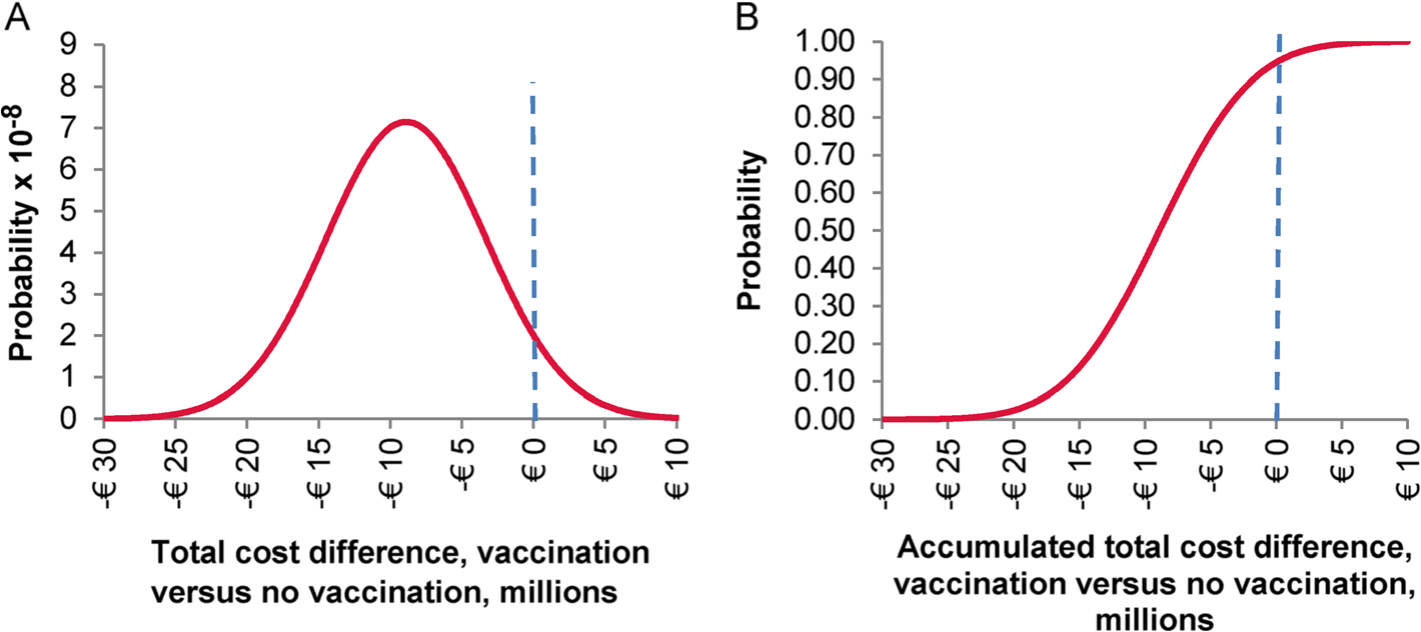
**Probabilistic sensitivity analysis results.** Results of the probabilistic sensitivity analysis, incremental cost (vaccination versus no vaccination. **A**: spread of the cost difference. **B**: cost density curve.

The results of the one-way and the probabilistic multi-way sensitivity analyses are shown in Figure 2 and Figure 3, respectively.

Figure 2 shows the effect of the different ranges of the input variables on the total cost difference (vaccination versus no vaccination), presented as a tornado diagram. Three factors were identified that could increase the incremental cost of vaccination above the cost-neutral point (i.e., the point at which vaccination would no longer be a cost-saving intervention). These factors were the frequency of seeking medical advice, frequency of rotavirus disease and frequency of hospital visits.

The overall result of the PSA first identifies the spread of results for the total cost difference. This spread of results follows a Normal distribution (Figure 3A). The density curve indicates that around 95% of the results would lead to an overall cost saving for vaccination compared with no vaccination (Figure 3B).

**Figure 4:**
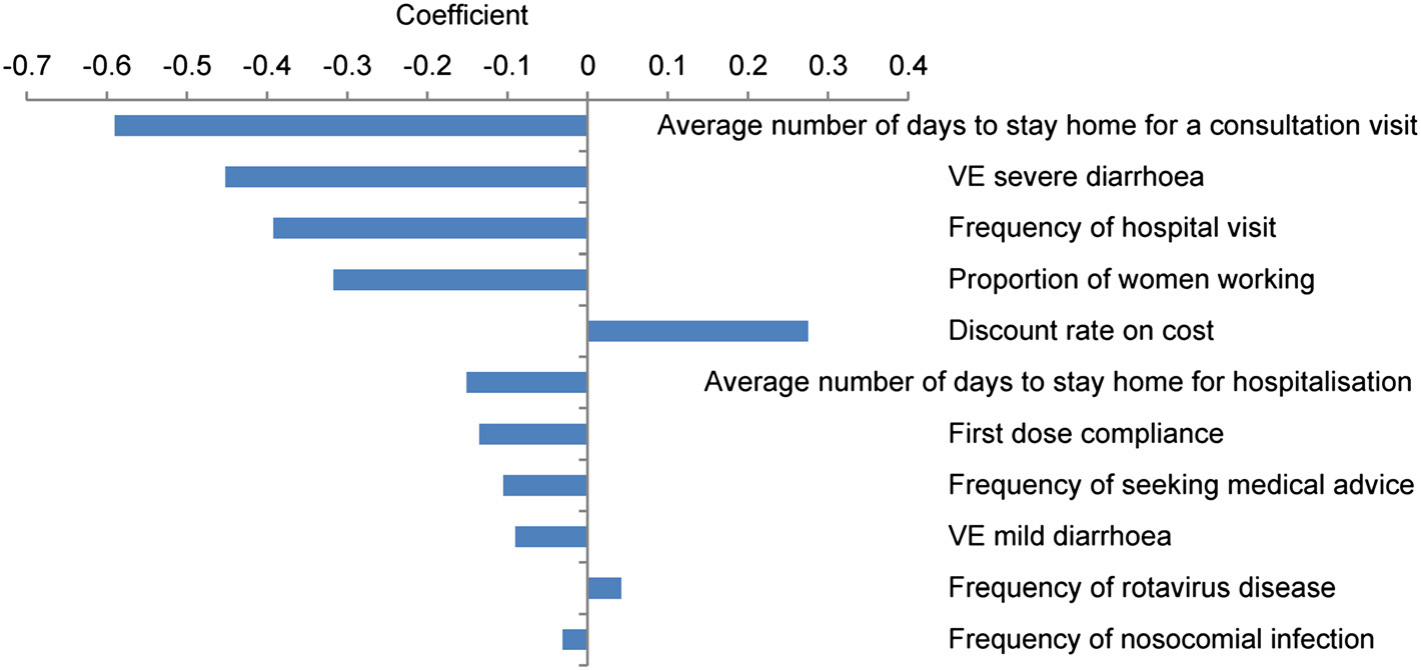
**Regression analysis.** Coefficients from the regression analysis on probabilistic sensitivity analysis results.

Analysis of the 5% of simulations that were not cost-saving showed that the results were essentially driven by a few factors: the number of days staying at home for RVGE, VE against severe disease and frequency of hospitalisation. This was also supported by the coefficient values from the regression analysis of the projected outcome results of the PSA (Figure 4).

## Discussion

The base-case results of the model presented here indicate that full vaccination with the two-dose RIX4414 vaccine in Germany has the potential to prevent almost 82% of RVGE cases in a birth cohort over the age range 0–5 years. As a consequence, rotavirus vaccination would be expected to produce considerable reductions in healthcare resource use, costs of hospitalisations and physician visits, and reimbursement of parents’ productivity losses because of their absence from work to care for a child with RVGE. The potential savings for the German SHI are projected to reach a total of €9.2 million for a birth cohort the size of that in 2008 with 100% vaccination coverage. With a more realistic vaccination coverage rate of 75%, the projected savings would be smaller but still substantial, at around €6.9 million.

Extensive sensitivity analyses were conducted to assess the effects of uncertainties in the epidemiological and cost data used in the model. The results of these analyses support the robustness of the base-case findings. They indicate that cost savings from rotavirus vaccination depend on a small number of key factors. The results underline the importance of the SHI reimbursement for productivity losses when parents stay at home to care for a sick child with RVGE. This reimbursement is a substantial cost, estimated at €18 million for the birth cohort over a 5-year period, and can be considerably reduced by vaccination. Other key drivers include high hospitalisation uptake and VE against severe disease requiring hospitalisation. It is interesting to note the differences between the one-way and multi-way sensitivity analyses; the latter illustrates how the different variables interplay in their effects.

Many cost-effectiveness analyses on rotavirus vaccination have been published for highly industrialised countries such as the UK [58], France [25,59], Belgium [60], the Netherlands [61], Italy [62], and the US [63]. The results of these analyses are influenced by a variety of factors such as the cost perspective selected (Ministry of Health or societal), the time horizon covered (3 years, 5 years or lifetime), and the input values chosen for health effects (e.g. QALYs), epidemiology and costs. Results may also be influenced by the organisation and structure of the healthcare system itself, such as the ease of access to hospital care (which may affect the hospitalisation rate), or whether absenteeism for work is reimbursed [23,64]. Concentrating on analyses from the perspective of SHI in Germany, the assumed frequency of rotavirus disease was a key driver for the results presented here and for the only other available analysis conducted from this perspective [24]. To the authors’ knowledge, the model published by Aidelsburger [24] does not correct the epidemiological data applied for unreported RVGE cases. The reimbursement of productivity losses due to paediatric diseases is an interesting feature of the German healthcare system that has not often been considered in cost analyses of rotavirus vaccination from the perspective of third-party payers. It is an important component of cost for the SHI and, as shown in the results presented here, it can be reduced substantially by vaccination. The cost savings from this reimbursement contribute substantially to reaching the cost-saving threshold for rotavirus vaccination. The SHI normally reimburses 70% of the net income of a parent staying away from work due to caring for a child with an illness, as well as direct medical costs. Obtaining SHI reimbursement requires a sickness certificate, but unfortunately there are no precise data available on reimbursement requests by disease type in children. In the modelling exercise described here, we assumed that all families in which both parents are employed will claim reimbursement from the SHI for caring for their sick child, and that no other reimbursement will be requested. We adopted a conservative estimate of 31% for the proportion of families with both parents working. This is based on the numbers of working mothers reported by the Federal Statistical Office of Germany for 2008. As this percentage is steadily rising over time, the impact on the SHI healthcare budget of sick leave due to rotavirus infection may increase in the future.

The model selected and the analysis performed have some limitations. The model was not designed to evaluate efficacy against individual rotavirus strains. It is known that the diversity of rotavirus strains circulating in the European population is high and varies by area and time, so it is possible that the results of the clinical trial from which we obtained VE data may not be appropriate for Germany. However, a high level of cross-reactivity against different circulating strains has been reported for the two-dose vaccine. Moreover, the sensitivity analysis indicates that the main driver of the cost results is the frequency of disease, with the VE range tested having a much smaller influence on the results.

It could be considered that we have omitted a reduction of VE over time, as seen in the European trial during the second year of observation. However, although this phenomenon is frequently suggested and reported as due to vaccine waning over time, it may be better understood as a process of increasing natural immunity over time in the control group. This would have the effect of reducing the difference between the vaccinated and unvaccinated groups in a trial as more time elapses. Observed data from Belgium after the introduction of rotavirus vaccination indicated no vaccine waning effect for the period studied (first four years after vaccine introduction) [65]. Therefore, the net difference in efficacy between the vaccinated and unvaccinated cohorts is measured and expressed over time in the model, and is not a process of vaccine waning.

Finally, our model is not a dynamic transmission model and so does not take account of any herd effects. It may therefore underestimate the effectiveness of vaccination against rotavirus when the coverage rate is less than 100%. Following the introduction of rotavirus vaccination in the US and a universal mass vaccination program in Austria, decreases in hospitalisations for rotavirus and gastroenteritis with no specified cause were observed among unvaccinated children and young adults (aged <24 years) [66,67]. In Belgium, rotavirus-related hospitalisations decreased after the introduction of rotavirus vaccination not only in the target age group for vaccination but also in unvaccinated children too young and too old to receive vaccination, indicating a herd protection effect [68]. In the US, it has been estimated that about 15% of the reduction in total hospitalisation and 20% of the reduction in direct medical cost attributable to rotavirus vaccination programmes occurred in unvaccinated 5–24 year-olds [66]. According to data from the Robert Koch Institut, the age group with the second highest number of rotavirus cases in Germany is the group aged 70 years and over (4600 rotavirus cases in 2012, compared with 19,299 in the age group 0–4 years) [69]. If herd effect could reduce the spread of rotavirus transmission to this age group, it has the potential to produce a sizeable reduction in the number of rotavirus cases in the elderly population in Germany. This could be an additional benefit of vaccination, but is not included in the model.

## Conclusions

Full vaccination against rotavirus in Germany with RIX4414 has the potential to prevent almost 82% of RVGE-related diarrhoea events in children aged 5 years and younger.

Rotavirus vaccination costs for the SHI could be fully offset by the cost avoided in hospitalisations, physician visits, and SHI-reimbursed productivity losses. With a more realistic vaccination coverage rate of 75%, vaccination would still achieve substantial cost savings for the SHI. Possible indirect effects of rotavirus vaccination, such as herd protection in age groups not targeted for vaccination (e.g. elderly people) and in unvaccinated individuals in the target age group when vaccine coverage is <100%, could further increase the potential for cost savings.

## Endnotes

^a^Rotarix is a registered trade mark of the GlaxoSmithKline group of companies.

^b^RotaTeq is a trademark of Merck & Co. Inc.

^c^Routine rotavirus vaccination was recommended by STIKO on 4 July 2013, after this manuscript was first submitted.

## Abbreviations

AGE: Acute gastroenteritis; DRG: Diagnosis-related group; EBM: Einheitlicher Bewertungsmaßstab; PSA: Probabilistic sensitivity analysis; RVGE: Rotavirus gastroenteritis; SHI: Statutory health insurance; STIKO: Ständige Impfkommission; VE: Vaccine efficacy.

## Competing interests

SK, CM and BS are employees of the GlaxoSmithKline group of companies, and BS holds stock in the GlaxoSmithKline group of companies. KV’s and UB’s institution received fees from the GlaxoSmithKline group of companies for participation in literature review, modelling, statistical analysis and for writing the initial version of the present manuscript.

## Authors’ contributions

SK and BS developed the model; CM, SK and KV reviewed the literature; SK, KV, UB and BS populated the model and determined the model settings; SK and KV acquired the data; BS and KV conducted the sensitivity analyses; BS, KV and UB provided statistical support and analysis; SK and KV drafted the manuscript; all authors provided scientific advice. All authors critically reviewed the study report. All authors reviewed and commented on drafts, read and approved the final manuscript.
